# Antibiotic Use in Relation with Psychological Profiles of Farmers of a French Pig Cooperative

**DOI:** 10.3390/vetsci9010014

**Published:** 2021-12-31

**Authors:** Jean-Charles David, Arnaud Buchet, Jean-Noël Sialelli, Sylvain Delouvée

**Affiliations:** 1 LP3C (Laboratoire de Psychologie: Cognition, Comportement, Communication), Department of Psychology, University Rennes, EA 1285, 35000 Rennes, France; jean-charles.david@univ-rennes2.fr; 2 Cooperl Innovation, Pôle Sciences Animales, Rue de la Jeannaie, 22403 Lamballe, France; arnaud.buchet@cooperl.com; 3 SELAS HYOVET, Carrefour de la Penthièvre, 22640 Plestan, France; jnsialelli@hyovet.com

**Keywords:** social psychology, antibiotics, livestock, psychological profiles, communication, change management

## Abstract

(1) Background: We focus on the psychological factors that influence pig farmers’ antibiotic use, which is not exclusively determined by the technical, health and structural factors of livestock farming. (2) Methods: We visited farming sites and asked 91 pig farmers about various psychosocial dimensions that could be considered relevant in explaining antibiotic use. (3) Results: The results indicate the existence of three livestock-farmer sub-profiles, each of which is associated with distinct psychological characteristics and antibiotic use levels. (4) Conclusions: We discuss the implications of antibiotic use for livestock in terms of communication and support.

## 1. Introduction

Over the past several decades, numerous health and environmental crises have shaken the livestock sector. African swine fever (ASF) and the proliferation of green algae in coastal areas are well-known examples. Today, the problem of antibiotic resistance is attracting attention—and for good reason [1]. Certain characteristics of intensive farming, such as the large number of animals and the close proximity among congeners, lead to significant antibiotic consumption [2]. In addition, many scholars have linked antibiotic use to the emergence of resistant bacteria [3,4], which in turn poses a risk to the health of both humans and animals. For example, global organizations such as the World Health Organization (WHO) and the World Organization for Animal Health (OIE) have encouraged farmers around the world to reduce antibiotic use. In 2011, the European Union embarked on a plan to control the public health risk associated with the use of antibiotics in veterinary medicine. In addition, the French Ministry of Agriculture’s Ecoantibio plan (2012–2017) addresses this issue; the first version of this plan had the objective of reducing antibiotic use in veterinary medicine by 25% in 5 years. In its 2018 report, the National Agency for Food, Environment and Occupational Health and Safety (ANSES) examined antibiotic sales in veterinary medicine and found a 37% decrease in all livestock animals’ exposure to antibiotics over this period; for pigs specifically, the decrease was 41.5%. Pig farms have benefited from the actions that the Ecoantibio plan set in motion, but pigs’ exposure to antibiotics remains high in comparison with that of other animals; pigs have an ALEA (animal level of exposure to antimicrobials) of 0.51, compared with 0.25 for cattle and 0.40 for poultry [5]. The second version of the Ecoantibio plan (2017–2021) aims to continue the momentum of the first version by consolidating achievements and continuing existing actions, such as communication and training on how to combat antibiotic resistance, development of measures to prevent infectious diseases, and facilitation of alternative treatments.

All the actors in the livestock sector are mobilized towards this goal, including veterinarians, farmers, agricultural groups and research organizations. Thus, in order to reduce antibiotic consumption, animal epidemiologists have devoted a whole range of research to identifying the factors that influence antibiotic use [6,7,8]. Among these factors, Lhermie et al. [9] highlight two. The first factor is exogenous variables such as weather and the seasons; infectious respiratory disorders are more common in winter, for example [10]. The second factor concerns endogenous variables; this refers, in particular, to the characteristics of individual farms and the behaviors of individual farmers. Endogenous factors are easier to control than exogenous ones, as both livestock practices and farms’ structural and technical aspects can be modified, but this is not the case for weather and seasons. Consequently, researchers are focusing on endogenous factors in order to identify actions that could reduce antibiotic use. Among these actions, farmers can implement biosecurity measures (e.g., work clothing, entrance locks and forwards marching) that are aimed at reducing the risks of infectious diseases being spread within farms or transmitted into them. The application of these measures helps farmers to reduce disease occurrence and to maintain their animals’ health statuses. For example, Hemonic et al. [11] showed that there is a large variability in antibiotic use between farms, due in part to the varying quality of their biosecurity. This study’s results indicate that antibiotic consumption is significantly lower on farms that have a good level of external biosecurity. Similar evidence is also reported in the literature regarding the positive role of improved biosecurity on antibiotic use [12,13,14]. The structural aspects of farms are also determinants of antibiotic use [15]. For example, overcrowding in livestock buildings and batch mixing are both associated with significant antibiotic use [16]. In terms of the animals’ housing conditions, buildings with ventilation and temperature management allow farmers to limit the occurrence of bacterial and viral diseases [17]. On the other hand, in recent years, European projects such as EFFORT or MINAPIG, have evaluated and identified agricultural strategies to reduce antimicrobial use while ensuring pig health and welfare and providing sustainable solutions for farmers. The resulting research has demonstrated, for example, that reduction is possible without compromising the technical and economic performance of farms [18,19].

These studies are examples of researchers’ growing interest in identifying and overcoming the obstacles to reducing antibiotic use. However, Chauvin et al. [20] showed that the factors that are traditionally used in animal epidemiology explain only 50% of the variability in antibiotic use by farmers. Hémonic et al. [21] also showed that these characteristics cannot fully explain the concentration in antibiotic-use rates, with 50% of the treatments administered in only 25% of the farms. Considering these results and the still-high rate of antibiotic consumption in the pig-farming industry, Hémonic et al. underlined the need for researchers to diversify their angles of approach so as to better understand and implement the demedication process (e.g., through improved communication and support for farmers).

The farmer plays an important role in the antibiotic administration process [22]. The veterinarian delegates this task to the farmer after diagnosing and prescribing the antibiotics. In the pig industry, research conducted jointly in four European countries (Belgium, France, Germany, Sweden) and focused on farmers’ perception of antibiotics showed that there are many common characteristics between countries [22,23]. The results highlight the poor perception by farmers of regulatory constraints or financial penalties (which would apply to heavy users) as a lever for change in usage. They better perceive support by the veterinarian, financial incentives (bonuses, premiums) and voluntary approaches. The problem of antibiotic resistance does not seem to be a major concern, even though farmers who are aware of this risk have a lower use than others. Another study [19] also highlighted the importance of compliance with veterinarians’ recommended actions, i.e., actual implementation of the prescribed treatment, to reduce use in swine production. This study also shows that farmers adhere better to proposals when they are convinced a priori of their effectiveness, and highlights the importance of the concept of “perceived control” described in rabbit farming [24].

### Aims of This Study

In this study, we aim to investigate the relationship between the psychological profile of farmers and their use of antibiotics in animal husbandry. To identify the psychological dimensions that may influence antibiotic use, we used a model of farmers’ behavior and decision making [25]. According to this model, farmers’ behaviors are influenced by personality traits, attitudes and goals. First, we measured farmers’ work motivation, as this dimension predicts workers’ performance, their need to learn new skills and to pursue new goals such as reducing antibiotic use [26]. Second, we measured farmers’ attitudes toward medicine and health, as the veterinarian is a preferred source of information for farmers when adopting new health practices [27]. We believe that a lack of trust in medicine or in one’s own veterinarian can be a barrier to following certain recommendations (e.g., antibiotic treatment or compliance with biosecurity measures) [28]. Finally, we measured locus of control, a personality trait that refers to how strongly people believe they have control over the situations and experiences that affect them [29]. People who believe that their performance depends primarily on themselves have an “internal” locus of control; those who believe the opposite (i.e., that the outcome is primarily determined by external factors beyond their influence) have an “external” locus of control. Therefore, this psychological dimension can be used to examine how a farmer is likely to explain an event in his work environment such as a bacterial contamination.

## 2. Study Design: Materials and Methods

### 2.1. Participants

This study’s sample consisted of 91 farmers from a cooperative in western France (Cooperl): 8 women and 83 men (M = 48.76; SD = 9.60, min = 27; max = 67). It is an agricultural cooperative specializing in the production of pigs and the processing of pork. The French situation is particular. More than 80% of French production is provided by farms located in the Grand Ouest region: Brittany (58%), Pays de la Loire (11%), Nouvelle-Aquitaine (7%), Normandy (6%). The chosen cooperative has existed since 1966 and the owners are the 2700 member breeders and produces 5,800,000 pigs each year. Therefore, choosing this cooperative ensures a certain representativeness in relation to French farms.

In our sample, the farms are all “Farrow-to-Finish”. The average number of sows on these farms was 249, which is slightly higher than the average for cooperative farms (237 sows). The average number of pigs produced per year on these farms was 5947 (about 6000 for all the farms in the cooperative). Finally, we ensured that our sample included farmers with different levels of antibiotic use: ALEA < 0.5 (59.85%), ALEA [0.5;0.9] (24.24%), ALEA > 0.9 (15.91%). Thus, there are more “heavy users of antibiotics” in our sample than in the cooperative as a whole: ALEA < 0.5 (75.10%), ALEA [0.5;0.9] (14.92%), ALEA > 0.9 (9.98%). These farmers have different veterinarians assigned from in the same veterinary company specialized in pig production. Diagnosis, prescription and delivery of antibiotics are carried out by this company for all farms considered.

### 2.2. Procedure

We used phone calls to make appointments for the participants to complete the questionnaire. During these calls, we specified the purpose of the questionnaire and the approximate time it would take to complete (30 min). A total of 132 breeders (owners of their farms) were contacted and 91 agreed to participate in the study (a response rate of 68.9%). We then administered the questionnaires from February through July of 2018; the participants completed the questionnaire in their own offices (in face-to-face with the investigator). The questionnaire consisted of 61 items that used a 7-point Likert scale (see Appendix A, Appendix B and Appendix C).

### 2.3. Measures

#### 2.3.1. Work Motivation 

We investigated the participants’ work motivation using the Work Extrinsic and Intrinsic Motivation Scale (WEIMS) [30], an 18-item scale based on that of Deci and Ryan [31]. The participants indicated their degree of agreement or disagreement using a 7-point Likert scale from 1 (*completely disagree*) to 7 (*completely agree*). The first factor refers to self-determined or intrinsic regulation, in which the sole motivation for an action is the interest and pleasure that an individual feels when carrying out that action (example item: “For the satisfaction I experience from taking on interesting challenges”). The second factor refers to extrinsic regulation, in which the activity is carried out not for pleasure but for reasons that are external to the individual, such as a financial reward or social pressure (example item: “Because it allows me to earn money”). The third and final factor refers to a complete lack of motivation (example item: “I don’t know, too much is expected of us”). McDonald’s omega coefficient indicates good scale consistency (𝜔 = 0.86).

#### 2.3.2. Attitude towards Medicine 

We based the measurement of the participants’ attitudes towards health and medicine on McFadden’s Complementary, Alternative and Conventional Medicine Attitudes Scale [32], which has a validated three-dimensional structure. The focus here is also on breeders. This questionnaire addresses the general view of medicine and health. We are then interested in the link between the conception of medicine and the possible treatments. The first dimension, philosophical congruence with complementary and alternative medicine, has 13 items, including “Treatments not tested in a scientifically recognized manner should be discouraged” (a reversed item). The second dimension, dissatisfaction with conventional medicine, has 6 items. The third dimension, holistic balance, was not used in this study; therefore, the version of the scale that the participants completed had 19 items. The participants indicated their degree of agreement or disagreement on a 7-point Likert scale from 1 (*completely disagree*) to 7 (*completely agree*). Compared with the scale’s initial validation, all the items were valid, with the exception of the Items 5 and 15; these two items did not saturate on the first dimension, so we removed them from future analyses. McDonald’s omega coefficient indicates good scale consistency (𝜔 = 0.81).

#### 2.3.3. Locus of Control 

To measure locus of control, we used the Levenson’s locus of control scale [33,34], which contains 24 items. As a psychological concept, *locus of control* refers to people’s tendency to see events as either controllable (internal locus of control) or uncontrollable (external locus of control). The questionnaire distinguishes external locus of control according to whether the control of events is attributed to luck or to another powerful person (e.g., a politician or line manager). McDonald’s omega coefficient indicates good scale consistency (𝜔 = 0.78).

#### 2.3.4. Indicators for Antimicrobial Consumption

The fourth selected indicator was the ALEA (*Animal Level of Exposure to Antimicrobials*), which is used by the national authorities to report on the yearly monitoring of antibiotic sales [35]. The ALEA value was calculated for each farm by the cooperative. It is calculated as follows: {[(quantities of active substance in mg)/(dose in mg/kg/d × duration in d)]/biomass in kg}.

(1) $$\mathrm{ALEA}=\frac{\mathrm{Weight}\;\mathrm{of}\;\mathrm{active}\;\mathrm{substance}\left(\mathrm{mg}\right)}{\frac{\mathrm{dose}\;\left(\mathrm{mg}\cdot {\;\mathrm{kg}}^{-1}\cdot {\mathrm{day}}^{-1}\right)\times \;\mathrm{treatment}\;\mathrm{lenght}\;\left(\mathrm{day}\right)}{\mathrm{biomass}\;\mathrm{at}\;\mathrm{risk}\;\mathrm{of}\;\mathrm{being}\;\mathrm{treated}\;\left(\mathrm{kg}\right)}}$$

### 2.4. Data Analysis

The farmers’ responses to the questionnaire were processed using IBM SPSS Statistics Version 25 [36]. To identify the psychological profile of the farmers, we conducted principal component analysis (PCA) using a hierarchical, bottom-up classification. To do this, we standardized the participants’ scores (between −1 and 1), calculated the distances between observations as Euclidean squared distances, and then aggregated the observations using Ward’s method [37,38]. Next, we determined whether it was possible to associate these psychosocial sub-profiles with the participants’ ALEA scores. We calculated the average ALEA for each cluster and compared these averages using a one-factor ANOVA. Finally, using the same statistical analysis, we determined the significance of the differences in the questionnaire scores between clusters. When the ANOVA was significant, post hoc comparisons were made with Fisher’s Low Significant Difference (LSD) test with a significance level of 0.05.

## 3. Results

### 3.1. Objective 1: Identify Participants’ Sub-Profiles According to Their Responses to the Questionnaires

**Figure 1:**
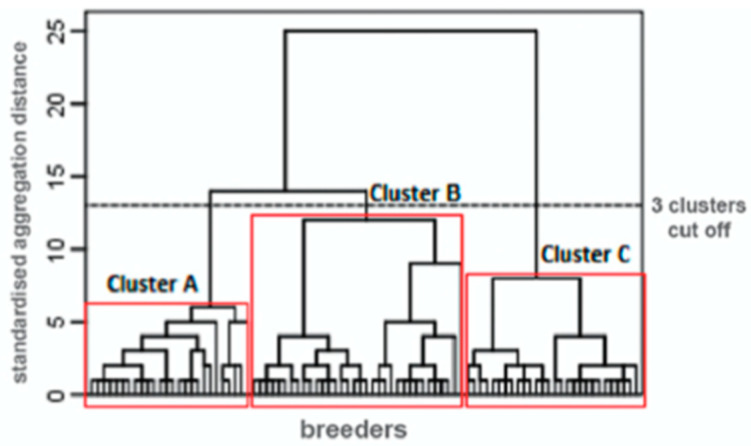
Dendrogram for a hierarchical cluster analysis on farmers’ questionnaire scores.

This study’s first objective was to verify whether the participants could be classified into distinct sub-profiles—that is, according to their responses to psychological questionnaires. The results of the hierarchical ascending classification are displayed in Figure 1. This analysis clearly revealed that certain subgroups of participants had distinct questionnaire scores. The classification with three sub-profiles (or “clusters”) was the most interpretable. The descriptive statistics for each cluster are presented in Table 1.

The clusters had significantly different average ALEA values (*F* = 4.03; *p* = 0.021). In addition, the results of least-significant difference tests show that, based on ALEA, the participants in Cluster A differ from those in Cluster B, but that those in Cluster C do not differ significantly from those in other clusters. Cluster A consists of those with low levels of antibiotic use (average ALEA = 0.37). Cluster B consists of those with high levels of antibiotic use (average ALEA = 0.65). Finally, Cluster C is characterized by moderate use (average ALEA = 0.51). The three clusters were comparable in terms of age (*F* = 1.43; *p* = 0.245) and sex (*χ*^2^ = 1.41; *p* = 0.494). 

### 3.2. Objective 2: Identify any Differences in Questionnaire Scores across the Clusters

This study’s second objective was to explore the clusters’ psychosocial dimensions. The average scores of each cluster for the various scales are presented in Table 2; the clusters differ significantly for most psychological variables (Table 3).

Of the three clusters, Cluster A (low ALEA) has the highest intrinsic-motivation score and the lowest amotivation score. Concerning health attitudes (the “convergence with complementary and alternative medicine” dimension), the Cluster A participants’ median scores are comparable with the scores of those in the other clusters. However, those in Cluster A trust conventional medicine more than those in Cluster B (high ALEA). In terms of locus of control, the participants in Cluster A have significantly lower scores than Cluster B for the “other powerful person” and “luck” dimensions. Concerning the participants’ internality, the median scores do not differ significantly across clusters. Cluster B (high ALEA) has the lowest average intrinsic motivation score of the clusters; Clusters B and C both have relatively high amotivation scores. Moreover, the participants in Cluster B have the highest dissatisfaction with conventional medicine and also have the highest scores for both the external dimensions of the locus of control (“other powerful person” and “luck”). Cluster C (moderate ALEA) is similar to Cluster A in terms of the psychosocial dimensions of attitudes towards medicine and locus of control. As stated above, Cluster C differs from Cluster A but is similar to Cluster B in terms of work motivation.

## 4. Discussion

This study shows a link between the psychological characteristics of farmers and the ALEA of their farm.

Reducing the use of antibiotics in the livestock sector requires the updating, or even the acquisition, of new skills. Work motivation predicts the functioning of individuals in organizations and their predisposition to modify their activity [39,40]. In particular, in the livestock sector, several studies have highlighted the role of motivation in decision making and behavior change (e.g., [41,42]). Our data show that intrinsically motivated farmers use the least amount of antibiotics. This type of motivation seems to support the initiative and innovation necessary to implement practices that reduce antibiotic use. In contrast, farmers in profiles B (high ALEA) and C (moderate ALEA) seem more amotivated. An amotivated worker feels that he or she is frequently in contact with situations that he or she cannot act on and escape. Thus, by ignoring the link between their husbandry practices and their antibiotic use, these amotivated farmers would be less likely to engage in behaviors that would reduce antibiotic use.

The results regarding locus of control support this. Farmers’ locus of control seems to have an impact on the health control of their farms. Indeed, in our study, we observe a relationship between farmers’ locus of control and ALEA. Farmers in profile C (high ALEA) are distinguished by a more external place of control than profiles A (low ALEA) and B (moderate ALEA). Thus, the farmer with an external locus of control would explain the occurrence of a pathology on his farm more by bad luck (e.g., difficult weather conditions) or by the fault of others (e.g., incompetent veterinarian). By this causal attribution, the farmer would not question the weight of his actions or the organization of his activity as, for example, the respect of biosecurity measures sometimes at the origin of the pathology. On the other hand, a farmer with an internal attribution of control will be more inclined to make the link between his behaviors and, for example, the appearance of a disease (as well as the emergence of antibiotic resistance and the perceived responsibility). He will, therefore, readjust some of his professional practices to remedy the situation.

Finally, the attitudes of the farmers regarding the vision of their own health seem to be transferred to breeding. The psychosocial definition of attitude is “a mental state that predisposes one to act in a certain way when the situation involves the real or symbolic presence of the object of attitude” [43]. Therefore, attitude is considered as an intermediate variable that prepares the individual to act in a certain way towards a given object, in our case, antibiotics and the means to reduce their use. Our results show that the “heavy users” of antibiotics were significantly less satisfied with “conventional” medicine compared with other farmers. Specifically, they were less convinced by the etiology of disease, more uncertain about the quality of physicians’ understanding of their health problems, and felt that physicians did not give them enough time. Therefore, we think that these attitudes are likely to impact the relationship between the farmer and the farm veterinarian. Indeed, veterinarians play an important role in supporting farmers since they prescribe antibiotics and are a source of health advice (vaccination, antibiogram) allowing a potential reduction in ALEA in farms [27,28]. In addition, reducing antibiotic use can be a source of stress for some farmers when this type of practice is perceived as a threat to the economic and health performance of the farm [24]. Thus, the relationship of trust between the veterinarian and the farmer may be a factor influencing compliance with recommendations to reduce antibiotic use in farm animals [44,45]. The latter, by establishing a relationship of trust, particularly through his / her expertise, can reduce uncertainties both in the health consequences of reducing antibiotic use and in the actions to be deployed to access it. Future research is needed to develop a scale to measure the trust relationship between the farmer and the veterinarian. Such an instrument would study the impact of this variable on compliance with recommendations (e.g., biosecurity measures) and drug treatments, and provide insight into how to improve the relationship between these two actors.

Our research has several limitations. One of the most important limitations is that our measures include only one indicator regarding antibiotic use on the farms surveyed. It would be appropriate to expand this measure by asking questions about specific husbandry practices, such as compliance with biosecurity measures. This would allow for a more detailed study of the relationship between psychological factors and the practices adopted by farmers to control the health status of their farms. Regarding measures, it would also be interesting to replicate this study by comparing the general view of human and animal medicine (e.g., by adapting the CACMAS).

Finally, our study did not consider the relationship between the farmer and the veterinarian, although this factor appears to influence antibiotic use [46]. Indeed, depending on the perceived relationship dynamics, the farmer–veterinarian relationship is a potential barrier or facilitator to antimicrobial use reduction. Identifying factors that positively influence collaboration between livestock producers and veterinarians could lead to shared responsibility for antimicrobial use reduction.

## 5. Conclusions

The results of this study show the influence of three psychological variables on antibiotic use in animal husbandry. Specifically, “heavy antibiotic users” differ from other farmers on several psychological dimensions, such as attitudes, breeding goals and personality traits, identified by Willock [25] as determinants of decision-making in animal husbandry. Therefore, it seems important to address the issue of reducing the use of antibiotics by activating certain psychological levers. For example, we think it is important to continue to educate farmers who use large amounts of antibiotics about their role in this process. These farmers need to realize that their daily behaviors have a direct impact on the health status of their farm and, therefore, on their use of antibiotics. However, some studies indicate that education is only possible if the farmer is open to receiving knowledge [19,47]. Therefore, it is necessary to reinforce this type of communication by training these farmers in simple, low-cost practices that have a rapid impact on health performance such as vaccination or hand hygiene (e.g., binding communication [48]). Proof of their effectiveness could encourage them, in a second phase, to make more profound changes in the organization of their activity (for example, by implementing the “marche en avant (The principle of “marche en avant” (forward motion) is the implementation of a qualitative approach to hygiene with the basic principle that healthy products should not cross the path of soiled products. This principle is mainly applied in catering but also in hospitals (Wikipedia).)“ on their farm). Here, again, the veterinarian or livestock technician can play a central role in gradually introducing these practices and breaking down the psychological barriers associated with them.

## Figures and Tables

**Table 1:** Descriptive statistics by cluster.

	N	Gender	Age	ALEA ª
Cluster A	28	27 men	46.21 (10.49) ^b^	0.37 (0.31)
Cluster B	34	30 men	49.52 (9.27)	0.65 (0.46)
Cluster C	26	24 men	50.31 (8.89)	0.51 (0.41)

ªALEA Animal Level of Exposure to Antimicrobials. ^b^ Mean (standard deviation in parentheses).

**Table 2:** Descriptive statistics of scores on scales by cluster.

	IM	A	CCAM	DMC	I	C	OA
Cluster A	6.02 (0.63) ^b^	2.23 (0.80)	4.82 (0.69)	2.51 (0.76)	4.81 (0.77)	3.04 (1.01)	2.72 (0.86)
Cluster B	5.24 (1.06)	4.26 (0.90)	4.66 (0.79)	3.29 (1.05)	5.12 (0.86)	3.68 (1.03)	4.19 (1.19)
Cluster C	5.47 (0.77)	4.47 (0.81)	5.02 (1.10)	2.52 (0.83)	4.82 (1.11)	2.65 (0.74)	2.77 (0.94)

^b^ Mean (standard deviation in parentheses). *IM* intrinsic motivation; *A* amotivation; *CCAM* convergence with complementary and alternative medicine; *DMC* dissatisfaction with conventional medicine; *I* locus of internal control; *C* locus of chance control; *OA* locus of control other almighty.

**Table 3:** Differences between the three clusters on the scales.

	*F*-Statistic	*p*-Value	*η·p*	Differences between Groups (LSD)
IM	6.65	**0.002**	0.14	A > B, C
A	61.32	**<0.001**	0.59	A < B, C
CCAM	1.24	0.295	0.03	N.S
DMC	6.24	**0.001**	0.15	A, C < B
I	1.91	0.324	0.03	N.S
C	16.40	**<0.001**	0.18	A, C < B
OA	43.64	**<0.001**	0.33	A, C < B

In bold the significant differences. N.S. means not significant. *IM*, intrinsic motivation; *A*, amotivation; *CCAM*, convergence with complementary and alternative medicine; *DMC*, dissatisfaction with conventional medicine; *I*, locus of internal control; *C*, locus of chance control; *OA*, locus of control other almighty.

## Data Availability

Data are available on request from the corresponding author.

## Appendix A. WEIMS (Original Version)

**Work motivation** ***Why Do You Do Your Work?***	**Dimension**
1. Because this is the type of work I chose to do to attain a certain lifestyle.	*Identified regulation*
2. For the income it provides me.	*External regulation*
3. I ask myself this question, I don’t seem to be able to manage the important tasks related to this work.	*Amotivation*
4. Because I derive much pleasure from learning new things.	*Intrinsic motivation*
5. Because it has become a fundamental part of who I am.	*Integrated regulation*
6. Because I want to succeed at this job;if not I would be very ashamed of myself.	*Introjected regulation*
7. Because I chose this type of work to attain my career goals.	*Identified regulation*
8. For the satisfaction I experience from taking on interesting challenges.	*Intrinsic motivation*
9. Because it allows me to earn money.	*External regulation*
10. Because it is part of the way in which I have chosen to live my life	*Integrated regulation*
11. Because I want to be very good at this work, otherwise I would be very disappointed.	*Introjected regulation*
12. I don’t know why, we are provided with unrealistic working conditions.	*Amotivation*
13. Because I want to be a “winner” in life	*Introjected regulation*
14. Because it is the type of work I have chosen to attain certain important objectives.	*Identified regulation*
15. For the satisfaction I experience when I am successful at doing difficult tasks.	*Intrinsic motivation*
16. Because this type of work provides me with security.	*External regulation*
17. I don’t know, too much is expected of us.	*Amotivation*
18. Because this job is a part of my life.	*Integrated regulation*

## Appendix B. CACMAS (Original Version)

**Complementary, Alternative, and Conventional Medicine Attitudes Scale**	**Dimension**
1. The health of my body, mind, and spirit are related, and whoever cares for my health should take them into account.	*Philosophical congruence with complementary and alternative medicine*
2. I have a more equal relationship with my complementary practitioner than with my doctor.	*Philosophical congruence with complementary and alternative medicine*
3. Effects of complementary therapies are usually the result of a placebo effect. (Reverse scored	*Philosophical congruence with complementary and alternative medicine*
4. I feel that complementary treatment is a more natural form of healing than orthodox medicine.	*Philosophical congruence with complementary and alternative medicine*
5. Complementary therapies are a threat to public health. (Reverse scored)	*Philosophical congruence with complementary and alternative medicine*
6. I feel so relaxed after complementary treatment sessions.	*Philosophical congruence with complementary and alternative medicine*
7. I believe that complementary medicine enables me to take a more active part in maintaining my health.	*Philosophical congruence with complementary and alternative medicine*
8. Most complementary therapies stimulate the body’s natural therapeutic powers.	*Philosophical congruence with complementary and alternative medicine*
9. Complementary therapies include ideas and methods from which conventional medicine could benefit.	*Philosophical congruence with complementary and alternative medicine*
10. Treatments not tested in a scientifically recognized manner should be discouraged. (Reverse scored)	*Philosophical congruence with complementary and alternative medicine*
11. I believe that complementary therapy will be more effective for my problem than orthodox medicine.	*Philosophical congruence with complementary and alternative medicine*
12. The explanation of my illness that I was given by my complementary practitioner made sense.	*Philosophical congruence with complementary and alternative medicine*
13.I value the emphasis on treating the whole person.	*Philosophical congruence with complementary and alternative medicine*
14. The last time I went to see a medical doctor, I was very satisfied with the care I received.	*Dissatisfaction with conventional medicine*
15. The last time I had important questions about my health care and I asked a medical doctor about them, I understood the answer. (Reverse scored)	*Dissatisfaction with conventional medicine*
16. I have a lot of confidence in the medical doctor I see most often for my health care. (Reverse scored)	*Dissatisfaction with conventional medicine*
17. I don’t trust doctors and hospitals, so I use them as little as possible.	*Dissatisfaction with conventional medicine*
18. The last time I saw a medical doctor, he or she did not understand my problem.	*Dissatisfaction with conventional medicine*
19. The last time I saw a medical doctor, he or she did not give me enough time.	*Dissatisfaction with conventional medicine*

## Appendix C. Locus of Control Scale (Originale Version)

**Item**	**Dimension**
1. Whether or not I get to be a leader depends mostly on my ability.	*Internal*
2. To a great extent my life is controlled by accidental happenings.	*Chance*
3. I feel like what happens in my life is mostly determined by powerful people.	*Powerful others*
4. My behavior will determine when I am ready to leave the hospital.	*Internal*
5. When I make plans, I am almost certain to make them work.	*Internal*
6. Often there is no chance of protecting my personal interests from bad luck happenings.	*Chance*
7. When I get what I want, it’s usually because I’m lucky.	*Chance*
8. Even if I were a good leader, I would not be made a leader unless I play up to those in positions of power	*Powerful others*
9. How many friends I have depends on how nice a person I am.	*Internal*
10. I have often found that what is going to happen will happen.	*Chance*
11. My life is chiefly controlled by powerful others	*Powerful others*
12. It is impossible for anyone to say how long I’ll be in the hospital.	*Chance*
13. People like myself have very little chance of protecting our personal interests when they conflict with those of powerful other people	*Powerful others*
14. It’s not always wise for me to plan too far ahead because many things turn out to be a matter of good or bad fortune	*Chance*
15. Getting what I want means I have to please those people above me.	*Powerful others*
16. Whether or not I get to be a leader depends on whether I’m lucky enough to be in the right place at the right time.	*Chance*
17. If important people were to decide they didn’t like me, I probably wouldn’t make many friends.	*Powerful others*
18. I can pretty much determine what will happen in my life.	*Internal*
19. I am usually able to protect my personal interests.	*Internal*
20. How soon I leave the hospital depends on other people who have power over me.	*Powerful others*
21. When I get what I want, it’s usually because I worked hard for it.	*Internal*
22. In order to have my plans work, I make sure that they fit in with the desires of people who have power over me.	*Powerful others*
23. My life is determined by my own actions.	*Internal*
24. It’s chiefly a matter of fate whether or not I have a few friends or many friends.	*Chance*
